# Early-Life Exposure to Malnutrition From the Chinese Famine on Risk of Asthma and Chronic Obstructive Pulmonary Disease in Adulthood

**DOI:** 10.3389/fnut.2022.848108

**Published:** 2022-05-31

**Authors:** Changbo Jin, Tiantian Zhang, Yongzhen Li, Wenming Shi

**Affiliations:** ^1^Shanghai First Maternity and Infant Hospital, Tongji University School of Medicine, Shanghai, China; ^2^School of Public Health, Fudan University, Shanghai, China; ^3^Department of Nutrition and Food Science, School of Public Health, Fudan University, Shanghai, China; ^4^School of Public Health, Li Ka Shing Faculty of Medicine, The University of Hong Kong, Hong Kong, Hong Kong SAR, China

**Keywords:** asthma, COPD, Chinese famine, peak expiratory flow, exposure window

## Abstract

**Objectives:**

Intrauterine malnutrition has a long-term effect on respiratory and lung function. However, few studies have explored the association between early-life exposure to famine with asthma and chronic obstructive pulmonary disease (COPD) in adulthood. Therefore, we aimed to investigate the association of early-life exposure to the Chinese famine of 1959–1962 with asthma and COPD later in life.

**Methods:**

This national population-based study included 6,771 participants from the baseline survey of the China Health and Retirement Longitudinal Study (CHARLS) who were born around the time of the Chinese famine. The famine exposure groups were determined according to the participants' birth year as non-exposed (1964–1967), fetal-exposed (1959–1962), preschool-exposed (1954–1957), and school-age exposed (1950–1953). Information about the demographic characteristics, self-reported doctor-diagnosed asthma and COPD, behavior and lifestyles, and indoor pollution were collected using validated questionnaires. In addition, peak expiratory flow (PEF) was measured to assess pulmonary function. Multivariable logistic regression and generalized linear mixed models were performed to explore the risk of adult asthma and COPD, PEF changes during various famine exposure periods compared with the non-exposed group. Stratified and sensitivity analyses were conducted to examine the modification and robustness of the association.

**Results:**

The prevalence of doctor-diagnosed asthma and COPD was 2.8 and 8.1%, respectively. Compared with the non-exposed group, the risk was significantly higher in the fetal-exposed group for asthma [adjusted odds ratio, (aOR) = 1.87, 95% confidence interval (CI):1.14–3.07] and the school-age exposed group [1.30 (1.00–1.69)] for COPD after controlling for confounders. Furthermore, we observed that fetal exposure to famine was significantly associated with a decrement of PEF in adulthood [β = −11.38 (−22.75 to −0.02)] compared with the non-exposed group. Stratified analyses showed that the association of asthma was stronger in men, who resided in severely famine-affected areas, smoked, and used solid fuels for cooking. No clearly consistent association was observed for subsequent COPD.

**Conclusions:**

Our results suggest that fetal exposure to the Chinese famine is significantly associated with the increased risk of asthma in adulthood. Future prospective studies are warranted to examine the association and mechanisms.

## Introduction

In recent years, the prevalence of chronic respiratory diseases has increased rapidly, resulting in a leading burden of non-communicable diseases (NCDs) globally (1). According to the Global Burden of Disease (GBD) study, chronic respiratory diseases were ranked as the third leading cause of death in 2017 (7.0% of all deaths) (2). Among them, asthma and chronic obstructive pulmonary disease (COPD) represent big health challenges of chronic respiratory diseases, which affected an estimated 358 million and 174 million individual worldwide in 2015, respectively (3). A national survey during 2012–2015 from the China Pulmonary Health (CPH) study reported that the prevalence of asthma and COPD was 4.2% and 8.6% in China, which posed a heavy burden on an individual's health and wellbeing (4, 5).

Peak expiratory flow (PEF), which used as an indicator of preliminary diagnosis and prognosis assessment of lung function and respiratory diseases including asthma and COPD, especially for older individuals (6). Growing evidence reported that exposure to an adverse environment, such as famine, malnutrition, and natural disaster during the critical development period, including *in utero*, early postnatal stage, or childhood had long-term implications for adult respiratory health (7, 8). Several studies indicated that malnutrition in early life, such as insufficient vitamin D level might reduce PEF and influence lung development (9, 10), while the mechanisms remained inconclusive. It is hypothesized that reducing in nutrition or oxygen supply has a range of adverse effects on cellular and molecular events in the developing lung, and can lead to structural changes that can persist into adulthood (11). An animal model also showed that severe malnutrition during early-life could promote the proliferation and effector functions of T helper 2 (TH2) cells, and DNA demethylation, which was associated with the susceptibility of lung diseases including asthma (12).

As one of the largest developing countries globally, China experienced a 3-year long famine between 1959 and 1962, which served as a “natural experiment” to explore the effects of early-life exposure to famine on health outcomes in adulthood. Previous studies have indicated that genes, dietary nutrients, smoking, alcohol drinking, and air pollution could influence the development of chronic respiratory diseases such as asthma (13–17); however, the evidence about the potential effect of early-life exposure to famine on subsequent asthma and COPD was limited. During the famine period, the limited economic status and extreme food scarcity affected many people, and fetuses experienced various degrees of malnourishment for a long time (18). Zhou et al. reported that early-childhood exposure to the Chinese famine might affect an individual's dietary habits, and the joint effect of famine and harmful dietary pattern could influence the health outcomes later in life (19). An epidemiological study in the Netherlands showed that exposure to Dutch famine (1944–1945) was significantly associated with the increased risk of later COPD and asthma hospitalization (20). In addition, a cohort study found that exposure to the Dutch famine in mid and early gestation related to the obstructive airways disease in adulthood (21). Compared with the Dutch Famine, the Chinese famine lasted for a longer time and was more severe in terms of extent. However, little was known about the long-term impacts of early-life exposure to famine on the risk of subsequent asthma and COPD and the changes of PEF among the Chinese adults.

In this study, we performed a national population-based study to investigate the effects of early-life exposure to the Chinese famine on the changes of PEF and associations with the risk of asthma and COPD in adulthood. Further, we aimed to explore the critical window of famine exposure that could serve as a reference for identifying the potential mechanisms of adult asthma and COPD and the vulnerability characteristics of the participants.

## Methods

### Study Design and Participants

This study used the baseline survey of the China Health and Retirement Longitudinal Study (CHARLS) between June 2011 and March 2012, a nationwide representative sample of middle-aged and older adults in China. Detailed design, sampling methods and data collection have been reported in prior literature (22). Briefly, this national survey covered 150 counties/districts and 450 villages/resident committees in China. A total of 17,708 respondents from 28 out of 31 provinces in mainland China (Tibet, Ningxia and Hainan were the exceptions) were included in the project. At each country or district unit, trained staff collected data based on a standard protocol in participants' homes and local health stations or office of the Chinese Center for Disease Prevention and Control (CDC). For each participant, the information about sociodemographic characteristics (such as age, gender, education, and marriage status), health status and biomarkers (such as doctor-diagnosed chronic diseases and blood testing), and community-level data (such as geographical area and public facilities) were collected by face-to-face computer-assisted personal interviews (CAPIs) with interviewers trained at Peking University by the CHARLS staff members. Ethics approval was obtained from the Biomedical Ethics Review Committee of Peking University (Nu: IRB00001052-11015). Written informed consents were obtained from all participants.

**Figure 1:**
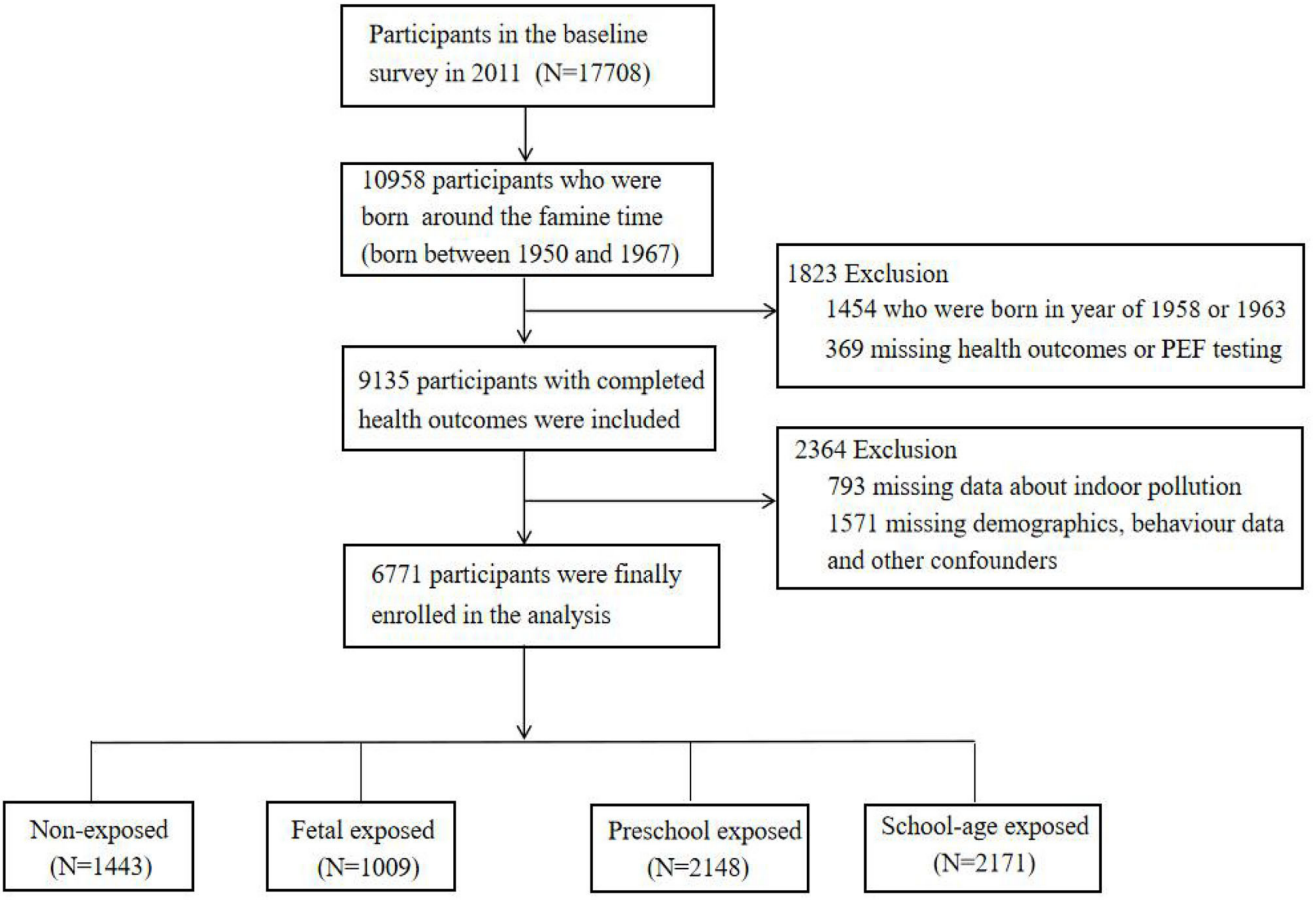
Flowchart of the participants enrollment.

Given that the Chinese famine happened during the period from 1959 to 1962, a total of 10,958 individuals who were born around the famine time (born between 1950 and 1967) from the CHARLS project were included. Since the exact start or end date of the Chinese famine was not clear, individuals who were born between January 1, 1958 and December 31, 1958 (*N* = 567) or January 1, 1963 and December 31, 1963 (*N* = 887) were excluded to minimize the misclassification bias. In addition, we excluded the participants who had missing data about health outcomes, PEF measurements, indoor pollution, and other confounders. A total of 6,771 participants were finally enrolled in our analyses. The flowchart of the participants' enrollment is shown in Figure 1.

### Assessment of Health Outcomes and PEF Measurement

Using the validated CHARLS baseline questionnaire, the self-reported doctor- diagnosed asthma or COPD were the interesting outcomes. They were evaluated using the question “Have you ever been diagnosed with asthma by a doctor?” or “Have you ever been diagnosed with the chronic obstructive pulmonary disease by a doctor?” (13). Participants who answered “Yes” were defined as suffering from the disease and vice versa.

PEF was tested by trained technicians using a peak flow meter with a disposable mouthpiece in units of L/min (Everpure™, Shanghai, China). Before the test, the technician demonstrated how the measurement was performed. The technicians instructed participants to stand up, take a deep breath and place their lips around the mouthpiece, and then conducted a quick and hard blow as best as possible. Interviewers recorded the readings indicated by the pointer and reset the meter for three measurements. The time interval between each measurement was set at 30 s by using a stop watch. The highest score among the three measurements was used in the analysis.

### Famine Exposure and Severity

Based on the previous studies about the Chinese famine (23, 24), we used the birth date as the proxy variable to assess famine exposure. All lunar birth dates were converted into solar birth dates firstly, all participants then were divided into four groups according to their birth dates: non-exposed (January 1, 1964 to December 31, 1967, used as the reference group), fetal-exposed (January 1, 1959 to December 31, 1962), preschool exposed (January 1, 1954 to December 31, 1957) and school-age exposed groups (January 1, 1950 to December 31, 1953). Similar to existing studies (23, 24), we used the excess mortality rate at the province-level to determine the severity of the Chinese famine. Excess mortality of 50% was set as a threshold value to classify the areas into severely or less severely affected areas. The excess mortality was calculated as the percentage of mortality rate changed from the mean level in 1956–1958 to the highest value during 1959–1961 (23, 24). The map of the distribution of the famine-affected areas in mainland China is shown in Supplementary Figure S1.

### Covariates

Individual information on sociodemographic characteristics, behavior and lifestyles, and indoor pollution were recorded by trained interviewers using a validated questionnaire. The sociodemographics included gender, age, body mass index (BMI), waist circumference (WC), educational level (≤primary school, middle school, and ≥high school), marriage status, family's financial status (≥average level vs. <average level, which was evaluated by the individuals' standard of living), and residential locations (urban vs. rural, which defined by the China's administrative divisions). In addition, behavior and lifestyle factors such as cigarette smoking status and alcohol consumption were collected. Cigarette smoking was assessed by asking the participants about whether they had the habit of smoking/ smoking a pipe or chewing tobacco, at present or in the past, and it could be classified into three groups (current; former; and never). Regarding alcohol consumption, individuals were asked using the question “Did you ever drink alcohol in the past? How often?” Only those who reported never drinking were defined as “No”. Indoor pollution from the type of household cooking fuels was determined using the question “What is the main source of cooking fuel?” Solid fuel was defined as a primary use of coal, liquefied petroleum gas, crop residue or wood-burning for cooking, while using electricity or natural gas was classified as clean fuels.

### Statistical Analysis

Descriptive analysis was applied to describe participants' sociodemographics, behavior, and lifestyle characteristics of participants according to the famine- exposure groups. The continuous variables were expressed as the mean ± standard deviations (*SD*) and categorical variables were described as counts and percentages, respectively. The Chi-squared tests or Analysis of Variance (ANOVA) were conducted to compare the characteristics of participants between groups. The prevalence of the self-reported diagnosed asthma and COPD was calculated across the different famine-exposure groups. Mann–Whitney *U*-test was performed to compare the difference of PEF indicator in each famine-exposure group with the non-exposed group.

Multiple logistic regression models were performed to investigate the association between early-life exposure to the Chinese famine and the risk of asthma and COPD in adulthood. A generalized linear mixed model was used to estimate the regression coefficients (β) and 95% CI for famine exposure on PEF changes. Both the crude and adjusted models were performed. The selection of the covariates used two criteria: (1) the well-known or possible risk factors of asthma or COPD based on the current knowledge; (2) variables changed the main effect ≥ 10% after included in the model (25). The covariates in this study included gender, BMI, WC, educational level, marriage status, family economic status, residential locations, cigarette smoking, alcohol consumption, and type of household cooking fuels. Multivariate analyses were fit to examine the robustness of association by including different covariates each time in the adjusted regression models.

To explore the effect modifier of several significant variables, stratified analyses were conducted to obtain the following: gender (men vs. women), the severity of famine-affected areas (severe vs. less severe), cigarette smoking (yes vs. no), and type of household cooking fuels (clean vs. solid).

Apart from the main analysis, we performed a sensitivity analysis: as the non-exposed group was younger than the other groups, we created a reference by merging the non-exposed and preschool-exposed groups as an age-balanced control. Moreover, we repeated our multivariate analysis to explore the association between famine exposures with outcomes compared with the control group.

All statistical analyses were conducted using STATA 16.0 (Stata Corp., College Station, TX, USA). A *p-*value <0.05 was considered as statistically significant.

## Results

A total of 6,771 participants were enrolled in this study; 3,067 (45.3%) were men. The sociodemographic characteristics of the participants according to the famine exposure groups are summarized in Table 1. Compared with the non-exposed group, participants who experienced famine exposure were more likely to be men, smoke, and use solid fuels for household cooking. We further compared the characteristics of the individuals who have diagnosed-asthma or COPD and the total participants. The descriptive statistics are presented in Supplementary Table S1.

**Table 1:** Sociodemographic characteristics and prevalence of asthma and COPD among the participants according to the famine exposure groups.

	**Non-exposed**	**Fetal-exposed**	**Preschool-exposed**	**School-age exposed**	***p*-value**
	**(*n* = 1,443)**	**(*N* = 1,009)**	**(*N* = 2,148)**	**(*N* = 2,171)**	
**Age in 2011 (years)**	44–47	49–52	54–57	58–61	–
**Men (%)**	579 (40.1)	450 (44.6)^a^	1,023 (47.6)^a^	1,015 (46.8)^a^	**<0.001**
**BMI (kg/m** ^**2**^ **)**	20.97 ± 6.98	20.98 ± 7.18	20.90 ± 7.24	20.63 ± 7.26	0.299
**Waist circumference (cm)**	84.71 ± 12.29	84.40 ± 13.51	84.51 ± 12.41	84.03 ± 12.88	0.672
**Educational level**					
≤ Primary school	745 (51.6)	490 (48.5)	1,393 (64.9)	1,678 (77.3)	**<0.001**
Middle school	512 (35.5)	277 (27.5)	479 (22.3)	354 (16.3)	
≥High school	186 (12.9)	242 (24.0)^a^	276 (12.8)	139 (6.4)^a^	
Married (%)	1,352 (93.7)	944 (93.6)	1,996 (92.9)	1,991 (91.7)	0.090
**Family's economic status**					
≥Average level	877 (60.8)	605 (60.0)	1,245 (58.0)	1,255 (57.8)	0.222
**<**Average level	566 (39.2)	404 (40.0)	903 (42.0)	916 (42.2)	
**Residential locations**					
Urban	596 (41.3)	386 (38.3)	786 (36.6)	816 (37.6)	**0.035**
Rural	847 (58.7)	623 (61.7)	1,362 (63.4)^a^	1,355 (62.4)^a^	
**Smoking status**					
Never	986 (68.3)	640 (63.4)	1,258 (58.6)	1,304 (60.1)	**<0.001**
Current	382 (26.5)	321 (31.8)	714 (33.2)	671 (30.9)	
Former	75 (5.2)	48 (4.8)^a^	176 (8.2)^a^	196 (9.0)^a^	
**Alcohol consumption**					
Yes	538 (37.3)	407 (40.3)	904 (42.1)	870 (40.1)	**0.040**
No	905 (62.7)	602 (59.7)	1,244 (57.9)^a^	1,301 (59.9)	
**Type of cooking fuels**					
Clean	478 (33.2)	321 (31.8)	624 (29.1)	620 (28.6)	**0.011**
Solid	965 (66.8)	688 (68.2)	1,524 (70.9)^a^	1,551 (71.4)^a^	
**Prevalence of diseases**					
Diagnosed asthma	29 (2.0)	38 (3.8)^a^	52 (2.4)	80 (3.7)^a^	**0.005**
Diagnosed COPD	97 (6.7)	71 (7.0)	165 (7.7)	217 (10.0)^a^	**0.001**

a *Indicated p < 0.05 compared with non-exposed group. The bold values indicate statistically significant*.

**Figure 2:**
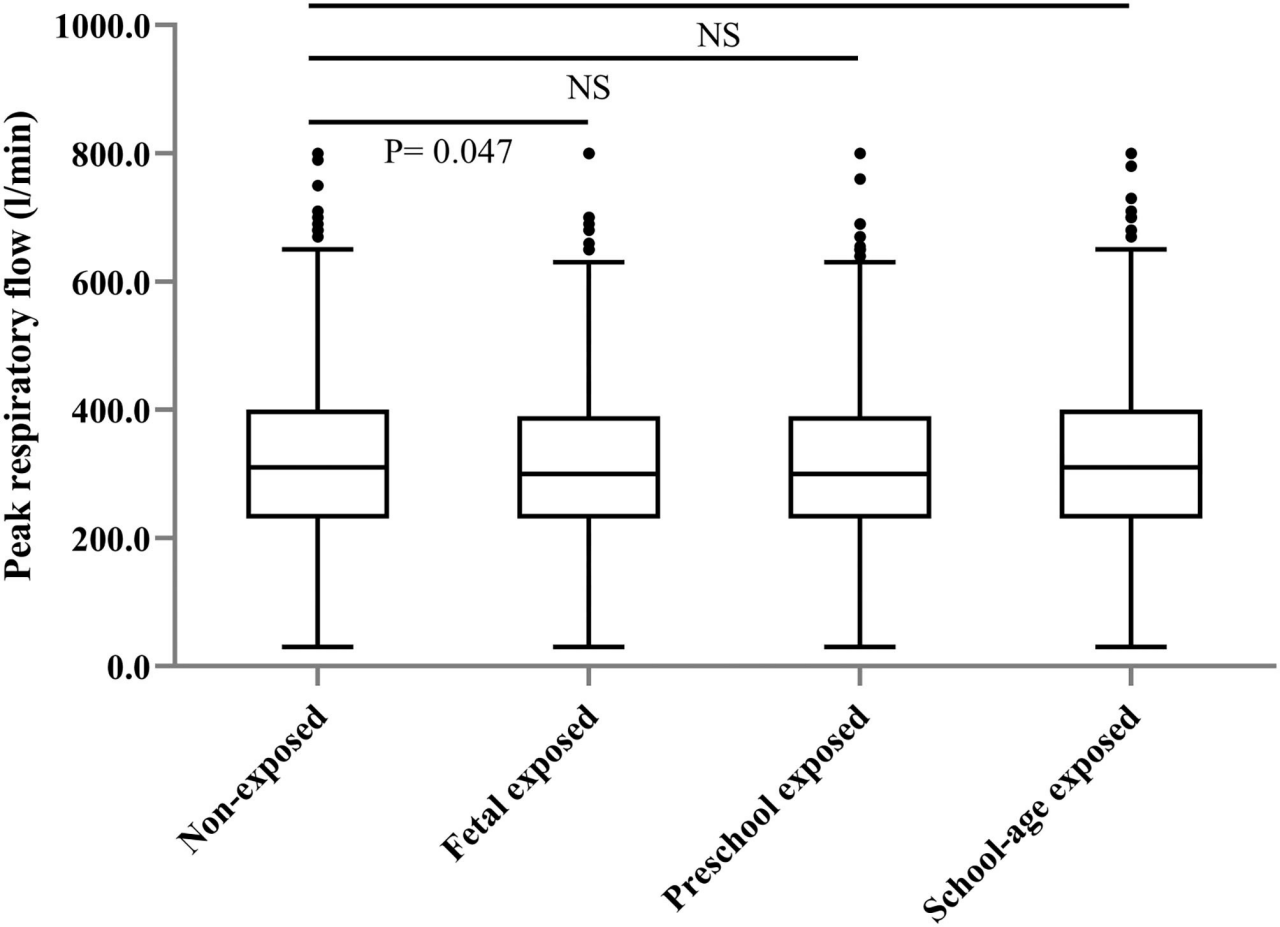
Distribution of peak respiratory flow level among the participants according to the different famine-exposure groups.

The prevalence of self-reported diagnosed asthma in the non-exposed group, fetal-exposed group, preschool-exposed group and school-age exposed groups were 2.0, 3.8, 2.4, and 3.7%, respectively. For COPD, the prevalence in each aforementioned group were 6.7, 7.0, 7.7, and 10.0% (Table 1). The value of PEF was significantly lower in the fetal-exposed group than that in the non-exposed group (*p* = 0.047) (Figure 2).

Compared with the non-exposed group, we found that the risk was significantly higher in the fetal-exposed group for asthma [adjusted odds ratio, (aOR) = 1.87, 95% CI: 1.14–3.07] and the school-age exposed group for COPD (aOR = 1.30, 95% CI: 1.00–1.69), respectively (Table 2, Model III). For PEF change, we observed that fetal-stage exposure to famine was significantly associated with a decrement of PEF in adulthood compared with the non-exposed group [β = −11.38 (−22.75, −0.02)] (Table 3, Model III).

**Table 2:** Crude and adjusted odds ratio for the association between early-life exposure to Chinese famine and risk of self-reported diagnosed asthma and COPD in adulthood.

	**Diagnosed asthma**		**Diagnosed COPD**	
	**OR (95% CIs)**	***p*-value**	**OR (95% CIs)**	***p*-value**
**Non-exposed**	1.00	Ref	1.00	Ref
**Fetal-exposed**				
Model I	1.91 (1.17–3.12)	**0.010**	1.06 (0.77–1.46)	0.708
Model II	1.88 (1.14–3.08)	**0.013**	1.05 (0.76–1.46)	0.750
Model III	1.87 (1.14–3.07)	**0.013**	1.05 (0.76–1.45)	0.775
**Preschool-exposed**				
Model I	1.21 (0.76–1.91)	0.420	1.16 (0.90–1.52)	0.243
Model II	1.04 (0.66–1.66)	0.858	1.07 (0.82–1.39)	0.639
Model III	1.04 (0.65–1.65)	0.880	1.04 (0.80–1.36)	0.759
**School-age exposed**				
Model I	1.91 (1.25–2.94)	**0.003**	1.56 (1.22–2.01)	**0.001**
Model II	1.50 (0.97–2.32)	0.073	1.32 (1.02–1.71)	**0.035**
Model III	1.49 (0.96–2.32)	0.076	1.30 (1.00–1.69)	**0.048**

*OR, odds ratio; 95% CIs, 95% confidence intervals; Ref, reference*.

*Model I: crude model*.

*Model II: adjusted for gender, BMI, WC, educational level, marriage status, family's financial status and residential locations*.

*Model III: further adjusted for smoking status, alcohol consumption and type of cooking fuels based on Model II*.

*The bold values indicate statistically significant*.

**Table 3:** Association between early-life exposure to Chinese famine and PEF indicator in adulthood.

**PEF change**	**Non-exposed**	**Fetal-exposed**	**Preschool-exposed**	**School-age exposed**
	**β (95% CI)**	**β (95*%* CI)**	**β (95% CI)**	**β (95% CI)**
Model I	Ref	−13.25 (−24.53, −1.98)*	−6.86 (−16.20, 2.48)	−6.23 (−15.54, 3.09)
Model II	Ref	−11.45 (−22.85, −0.05)*	−6.29 (−15.79, 3.21)	−4.72 (−14.33, 4.88)
Model III	Ref	−11.38 (−22.75, −0.02)*	−6.19 (−15.71, 3.33)	−4.63 (−14.26, 4.99)

*95% CI, 95% confidence intervals; Ref, reference*.

*Model I: crude model*.

*Model II: adjusted for gender, BMI, WC, educational level, marriage status, family's financial status and residential locations*.

*Model III: further adjusted for cigarette smoking, alcohol consumption and type of cooking fuels based on Model II*.

*indicated p-value < 0.05.

Stratified analyses showed that men and those who lived in severely famine-affected areas had a higher risk of adult asthma (Table 4). In men, when compared with the non-exposed group, school-age exposure to the Chinese famine significantly increased the risk of asthma (aOR = 2.48, 1.24–4.99) later in life. Regarding famine severity, we observed a significantly higher risk of asthma in the fetal-exposed group (aOR = 2.08, 1.01–3.52) compared with the non-exposed group. After stratification by cigarette smoking and type of household cooking fuels, stronger associations for adult asthma and COPD were observed in individuals who smoked or used solid biomass fuels at home (Table 4). Compared with the non-exposed group, those who smoke have a higher risk of asthma (aOR = 2.15, 1.38–3.04) when fetal exposed to famine. Moreover, those who use solid fuels showed a risk of asthma and COPD in adulthood in the fetal-exposed and the school-aged exposed groups, respectively (Table 4). For PEF indicator, the multivariate analyses showed that significant associations in those who living in severely famine-affected areas and using solid fuels when the fetal exposed to famine (Supplementary Table S2). Sensitivity analyses by merging the non-exposed and preschool exposed groups as a reference group, we obtained very similar results (Supplementary Table S3). The aOR and 95% CIs were 1.83 (1.23–2.73) for the fetal-exposed group (for asthma), 1.48 (1.07–2.04) for asthma and 1.26 (1.04–1.54) for COPD in the school-age exposed group (Supplementary Table S3).

**Table 4:** Adjusted OR and 95% CI of the association between famine exposure with asthma and COPD stratified by potential modifiers (reference: non-exposed group).

**Variables**	**Categories**	**Exposure**	**Adjusted OR (95% CIs)**	
			**Asthma**	**COPD**
Gender	Men	Fetal-exposed	1.95 (0.85–4.43)	1.33 (0.85–2.06)
		Preschool-exposed	1.27 (0.60–2.69)	1.02 (0.69–1.51)
		School-age exposed	2.48 (1.24–4.99)*	1.43 (0.99–2.07)
	Women	Fetal-exposed	1.05 (0.84–1.20)	0.80 (0.49–1.31)
		Preschool-exposed	0.93 (0.51–1.70)	1.06 (0.73–1.53)
		School-age exposed	0.95 (0.52–1.73)	1.21 (0.84–1.75)
Severity of famine-affected areas	Severe	Fetal-exposed	2.08 (1.01–3.52)*	1.21 (0.78–2.13)
		Preschool-exposed	1.32 (0.59–2.96)	1.15 (0.81–1.83)
		School-age exposed	1.69 (0.77–3.69)	1.37 (0.99–1.89)
	Less severe	Fetal-exposed	1.25 (0.64–2.45)	1.26 (0.75–2.10)
		Preschool-exposed	0.92 (0.52–1.63)	1.20 (0.77–1.88)
		School-age exposed	1.44 (0.84–2.45)	1.20 (0.76–1.87)
Cigarette smoking	Have	Fetal-exposed	2.15 (1.38–3.04)**	1.25 (0.82–1.89)
		Preschool-exposed	1.09 (0.52–1.63)	1.12 (0.66–1.37)
		School-age exposed	1.86 (0.72–2.32)	1.34 (0.94–1.89)
	Have not	Fetal-exposed	1.04 (0.41–2.68)	0.80 (0.48–1.35)
		Preschool-exposed	1.19 (0.56–2.52)	0.95 (0.78–1.71)
		School-age exposed	1.48 (0.95–2.78)	1.32 (0.89–1.96)
Type of cooking fuels	Clean fuels	Fetal-exposed	1.86 (0.68–3.05)	1.13 (0.61–2.10)
		Preschool-exposed	0.81 (0.30–2.23)	0.85 (0.49–1.47)
		School-age exposed	1.48 (0.60–3.64)	1.01 (0.60–1.70)
	Solid fuels	Fetal-exposed	1.85 (1.05–3.27)*	0.98 (0.67–1.45)
		Preschool-exposed	1.10 (0.65–1.87)	1.09 (0.80–1.49)
		School-age exposed	1.51 (0.91–2.50)	1.41 (1.05–1.91)*

*p < 0.05,

**p < 0.01.

## Discussion

In this study, early-life exposure to the Chinese famine was significantly associated with a decreased PEF and increased risk of diagnosed asthma in adulthood. Our results suggested that the fetal stage might be a critical period to the growth and development of lung functions. Nevertheless, we have not observed consistent results about school-age exposure to famine with COPD. Men, those living in the severely famine-affected area, and smoked might have a higher risk of subsequent asthma. Further, the association of both asthma and COPD was pronounced in those who used biomass solid fuels for cooking at home. However, no significant association was observed between preschool exposed to famine with asthma and COPD in later life.

Similar to a previous study (21), we found that exposure to the Chinese famine during the fetal stage was significantly associated with the risk of asthma in later life. van Abeelen et al. found that individuals who experienced the Dutch Famine during 1944–1945 increased the risk of later asthma and COPD hospitalization (20). A cross-sectional study in China showed that infant-exposure to severe famine was positively related to the elevated risk of chronic lung diseases among male adults (26). Another prospective study based on the China Kadoorie Biobank (CKB) project found that prenatal exposure to famine was not significantly associated with the risk of respiratory diseases such as COPD (27). Taken together, the sample size, study design and potential confounders might contribute to these contradictory results. Our study evaluated the association between famine exposure and PEF indicator. The significant results further demonstrated that fetal-stage exposure might be a critical window. Despite these, future cohort studies are still needed to be replicated to validate this finding.

Compared with the Dutch Famine during 1944–45, the Chinese famine was more severe with higher mortality (28). Most participants suffered from the unfavorable effects of food deficiency and malnutrition during this period. Malnutrition in an early life could lead to intrauterine growth restriction (IUGR) and low birth weight, which was associated with an increased risk of asthma from childhood to adulthood (29). A recent epidemiological study demonstrated that IUGR increased the risk of asthma continuously from the ages of 7–43 years (30). Furthermore, some studies indicated that vitamin D deficiency in the fetal stage might adversely affect on the developing lung and immune system, and subsequently influencing lung function, and risk of asthma in later life (31, 32). Additionally, vitamin A has been essential in the normal development and differentiation of lung airways, and vitamin A deficiency is positively related to a lower PEF value and lung problems, including asthma (33). PEF is widely used as a risk assessment tool for lung function, because of its simple and readily available measurements (34). Similar to previous findings (10, 32), we observed that fetal exposure to malnutrition from famine was significantly associated with a decrement of PEF in midlife. There are several underlying biological mechanisms. According to the developmental programming of health and disease (DOHaD) hypothesis, the fetal stage is a period of rapid growth, any stimulus during this critical period can permanently alter or “programme” the structure and physiology of the respiratory system with possible long-term consequences (35, 36). Another possible mechanism could be that epigenetic changes modify bronchial reactivity and induce alternative phenotypes from the same genotype (7). Animal models showed that dietary protein restriction and malnutrition during early-life could lead to lower MeDNA levels in promoter regions of the glucocorticoid receptor and peroxisomal proliferator-activated receptor genes in mice offspring, which lead to persistent phenotype changes that could be transmitted to the next generation (37, 38).

The school-age years may represent a period of increased vulnerabilities of environmental exposure, because of the ongoing development of airways and alveoli and the high proportion of lung surface area relative to the child's overall size (39). Furthermore, school-age children often spend more time away from their parents at schools and other extracurricular activities, thus increasing the exposure opportunities. In this study, though we observed that the school-age exposure to the Chinese famine was significantly related to the elevated risk of subsequent COPD, the association for PEF changes was not significant. The possible explanations for these inconsistent results might be ascribed to the measurements of different outcomes and potential confounding in the study.

In parallel with a previous study (26), we observed the association of asthma was stronger in men and those residing in severely famine-affected areas. The possible reason behind this was that the development of male's lungs might be more vulnerable to severe malnutrition in early life. Although animal studies have reported that male fetuses have a higher risk of adverse effects than women, female fetuses may be less susceptible in adverse utero environment (40). It is reported that the placenta of female fetuses had a higher 11β-hydroxysteroid dehydrogenase type 2 (11- HSD2) level and lower level of maternal glucocorticoids than men. Furthermore, fetal exposure to glucocorticoids is a proposed mechanism leading to malnutrition, and it might be the underlying reason for the reduced respiratory system (41).

In this study, the stratified analyses indicated that smoking enhanced the risk of asthma in midlife after exposure to famine, consistent with existing studies (15, 42). Van Abeelen et al. (20) also found a dose-response relationship between famine exposure and the risk of computed tomography (CT) evidence of pulmonary disease including COPD among ever-smokers, while no significant association was observed among non-smokers. A prospective study following more than 2,500 children from the age of 7–53 years indicated that parental smoking during childhood was a determinant of low-lung function trajectories in adulthood, development of COPD, and overlap of asthma-COPD (43). For indoor pollution, we found that participants who used solid fuels for household cooking had stronger adverse effects, in line with previous studies (44, 45). Biomass smoke from cooking can increase the risk of adult asthma and reduce the lung function, especially in rural areas in developing countries (46, 47). Indoor burning of biomass fuels, which generates both particulate matter and gaseous pollutants, is associated with low birth weight or preterm birth, and increases the risk of childhood respiratory infections and lung development (44). Furthermore, a study showed that solid fuel smoke could lead to oxidative stress by increasing reactive oxygen species (ROS), which directly damaged DNA and promoted lung cell proliferation, and might result in reduced PEF and lung diseases (48).

To our knowledge, this was one of the large-scale surveys to explore the early-life exposure to Chinese famine on the risk of PEF indicator, and associations with asthma and COPD in later life. The findings provide important implications for mitigating the adverse effects of early-life famine exposure and promoting lung health in adults by reducing malnutrition. Furthermore, the nationally representative sample of the Chinese adults and comprehensive information on potential confounders were collected in our study, which provided more valid results. Third, a series of stratified analyses were performed in the study, which helped distinguish the critical exposure window in which famine exerts adverse effects on respiratory health and identify the potentially vulnerable characteristics.

This study has several limitations. First, similar to other famine studies, the selection bias cannot be avoided due to severe famine that could eliminate weaker participants and only the healthier participants remain. This may mitigate the real effect of famine exposure. Second, the assessment of asthma and COPD outcomes was based on self-reported questionnaires and the actual age of diagnosis was not recorded, which might introduce recall bias. However, it is difficult to measure the diseases using clinical diagnosis for each participant in such a large-scale study. In addition, the significant effects of Chinese famine exposure on PEF change, a biomarker of lung function, further validate our results. Due to the lack of the data of other related indicators, such as forced expiratory volume in 1 s (FEV_1_) and forced vital capacity (FVC), restricted our ability to analyze these relationships. Third, the objective indicators reflecting the severity of famine exposure, such as birth weight or birth body length, were not available in the study, which might cause misclassification. Fourth, limited by the current design and the lack of information about individuals' residential addresses, which does not allow us to control for the ambient air pollutants in the study, despite it might be a possible driver. Lastly, the potential confounding like family history of asthma and food allergy, immune system functions, dietary pattern, and physical activity was not adjusted in the model due to the lack of information. For all these limitations mentioned above, the findings should be interpreted with the caution before replicated in other famine cohort studies in the future.

## Conclusions

Our results suggest that fetal exposure to the Chinese famine may be associated with a decrement of PEF and an increased risk of asthma in later life, particularly in males and those living in severely famine-affected areas. This association is pronounced in those who smoke and use solid biomass fuels for cooking. However, no consistent association was observed between early-life exposures to famine with subsequent COPD. Hunger and food shortage remains evident in many countries, resulting in different manifestations of malnutrition and increasing disease burden. Our findings identify the long-term impacts of malnutrition from famine exposure in early life, as well as the vulnerable characteristics, which provide important public health implications. It is meaningful to take active intervention measures for individuals who experienced malnutrition during fetal stage to prevent and reduce the risk of asthma several decades later. The future prospective studies are warranted to validate our findings and explore the potential mechanisms.

## Data Availability Statement

The raw data supporting the conclusions of this article will be made available by the authors, without undue reservation.

## Ethics Statement

The studies involving human participants were reviewed and approved by Biomedical Ethics Review Committee of Peking University. The patients/participants provided their written informed consent to participate in this study.

## Author Contributions

WS designed the study. CJ, TZ, and WS collected the data and wrote the manuscript. CJ and TZ analyzed the data. WS and YL edited the manuscript. All authors were involved in writing the paper and had final approval of the submitted and published versions.

## Funding

This work was supported by the Development Research Center of Shanghai Municipal People's Government (No: 2021-S-13).

## Conflict of Interest

The authors declare that the research was conducted in the absence of any commercial or financial relationships that could be construed as a potential conflict of interest.

## Publisher's Note

All claims expressed in this article are solely those of the authors and do not necessarily represent those of their affiliated organizations, or those of the publisher, the editors and the reviewers. Any product that may be evaluated in this article, or claim that may be made by its manufacturer, is not guaranteed or endorsed by the publisher.
