# Supplementary material for: Early-Life Exposure to Malnutrition From the Chinese Famine on Risk of Asthma and Chronic Obstructive Pulmonary Disease in Adulthood
Source: Front Nutr. 2022 May 31;9:848108. doi: 10.3389/fnut.2022.848108 (PMC9194571; doi:10.3389/fnut.2022.848108)
Supplement: Supplementary file 1 [file Data_Sheet_1.PDF]

Supporting information

Early-life exposure to malnutrition from the Chinese famine on risk of asthma and chronic obstructive pulmonary disease in adulthood

Content

Figure S1. The map about the distribution of the famine-affected areas in China.

Table S1. Sociodemographic and behaviour characteristics of the participants who having asthma or COPD and the total participants in the study.

Table S2. Adjusted β and 95%CI of the association between famine exposure with PEF change stratified by potential modifiers (reference: non-exposed group).

Table S3. Adjusted odds ratio and 95% confidence interval for the risk of asthma or COPD by exposure to the Chinese Famine with combined old and young reference group.

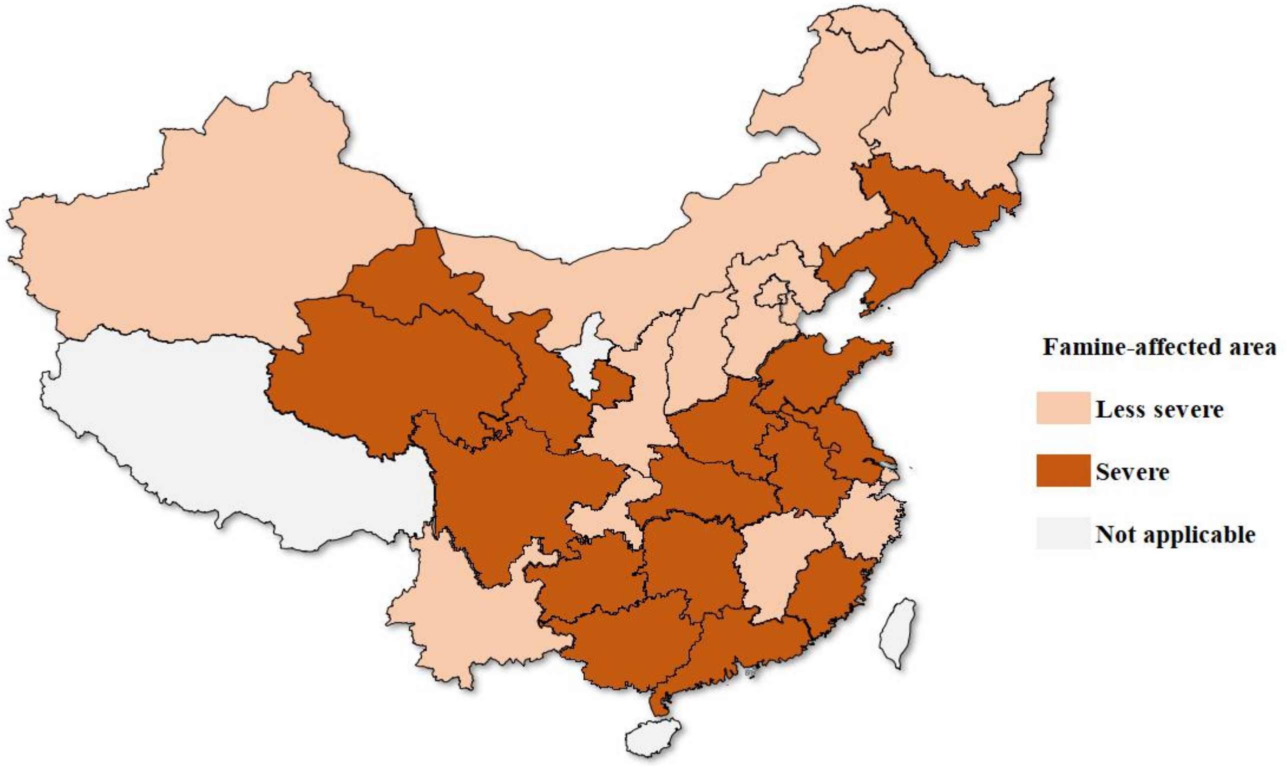

Figure S1. The map about the distribution of the famine-affected areas in China.

Table S1. Sociodemographic and behaviour characteristics of the participants who having asthma or COPD and the total participants in the study.

	Total (N=6771)	Diagnosed asthma (N=201)	Diagnosed COPD (N=550)
**Age (Mean ± Sd)**	54.12 ±5.11	55.04 ±4.92	55.0±5.0
**Male (%)**	3067 (45.3)	100 (49.8)	285 (51.8)
**BMI (kg/m^2)**	20.84 ±7.18	21.10 ±7.65	20.85 ±7.14
**Waist circumference (cm)**	84.38 ±12.70	84.99 ±11.55	84.41 ±12.77
**Educational level**			
≤ Primary school	4306 (63.6)	148 (73.6)**	396 (72.0)***
Middle school	1622 (24.0)	41 (20.4)	113 (20.5)
≥ High school	843 (12.4)	12 (6.0)	41 (7.5)
**Married (%)**	6307 (93.1)	179 (89.1)	505 (91.8)
**Family's economic status**			
≥ Average level	3982 (58.8)	111 (55.2)	278 (50.5)**
< Average level	2789 (41.2)	90 (44.8)	272 (49.5)
**Residential locations**			
Urban	2835 (41.9)	74 (36.8)	181 (32.9)
Rural	3936 (58.1)	127 (63.2)	369 (67.1)**
**Cigarette smoking status**			
Never	4188 (61.9)	117 (58.2)	287 (52.2)***
Current	2088 (30.8)	66 (32.8)	198 (36.0)
Former	495 (7.3)	18 (9.0)	65 (11.8)
**Alcohol consumption**			
Yes	2719 (40.2)	82 (40.8)	229 (41.6)
No	4052 (59.8)	119 (59.2)	321 (58.4)
**Type of cooking fuels**			
Clean	2043 (30.2)	45 (22.4)	130 (23.6)
Solid	4728 (69.8)	156 (77.6)*	420 (76.4)***

$^{*}P$ <0.05, ** P<0.01, $^{***}P$<0.001

Table S2. Adjusted β and 95%CI of the association between famine exposure with PEF change stratified by potential modifiers (reference: non-exposed group).

Variables	Categories	Exposure	PEF indicator
Gender	Male	Fetal exposed	-12.255(-27.087, 2.577)
		Preschool exposed	-11.174 (-25.646, 2.126)
		School-age exposed	-4.567 (-16.989, 8.076)
	Female	Fetal exposed	-9.405(-27.278, 8.468)
		Preschool exposed	-2.481 (-12.332, 9.239)
		School-age exposed	-3.388 (-18.482, 11.704)
Severity of famine-affected areas	Severe	Fetal exposed	-14.181(-25.298, -3.072)*
		Preschool exposed	-14.308 (-19.653,1.036)
		School-age exposed	-0.929 (-16.575, 14.717)
	Less severe	Fetal exposed	-7.539 (-22.342, 7.264)
		Preschool exposed	-1.978 (-14.075, 10.118)
		School-age exposed	-5.939(-18.129, 6.250)
Cigarette smoking	Have	Fetal exposed	-10.258(-32.532, 12.015)
		Preschool exposed	-0.749 (-19.310, 17.812)
		School-age exposed	-6.934(-26.045, 12.175)
	Have not	Fetal exposed	-10.412(-24.255, 3.431)
		Preschool exposed	-8.172(-19.883, 3.539)
		School-age exposed	-2.953(-14.705, 8.799)
Type of cooking fuels	Clean fuels	Fetal exposed	-2.517(-23.271, 18.236)
		Preschool exposed	-3.484(-21.240, 14.272)
		School-age exposed	-2.936 (-11.948, 7.821)
	Solid fuels	Fetal exposed	-10.412 (-19.255, -1.012)*
		Preschool exposed	-8.172 (-17.883, 1.539)
		School-age exposed	-2.952 (-14.705, 8.799)

*P <0.05

Table S3. Adjusted odds ratio and 95% CI for the risk of asthma or COPD in adulthood by exposure to the Chinese famine with combined old and young reference group.

	Diagnosed asthma	Diagnosed COPD
Non-exposed and preschool exposed	ref	ref
Fetal exposed	**1.83 (1.23–2.73)****	1.02 (0.77–1.35)
School-age exposed	**1.48 (1.07–2.04)***	**1.26 (1.04–1.54)***

Note : 95%CI：95% confidence intervals
Model adjusted for age, sex, BMI, WC, education, marriage status, cigarette smoking, alcohol consumption, family's financial status, residential locations and type of cooking fuels.

**P* <0.05,　***P* <0.01
